# Antibacterial and anti-inflammatory effects of *Syzygium jambos* L. (Alston) and isolated compounds on acne vulgaris

**DOI:** 10.1186/1472-6882-13-292

**Published:** 2013-10-29

**Authors:** Richa Sharma, Navneet Kishore, Ahmed Hussein, Namrita Lall

**Affiliations:** 1 Department of Plant Science, Faculty of Agricultural and Biological Science, University of Pretoria, Pretoria 0002 South Africa; 2 Chemistry Department, University of Western Cape, Private Bag X17, Bellville, 7535 Cape Town, South Africa

**Keywords:** *Syzygium jambos*, *Propionibacterium acnes*, Antibacterial, Interleukin 8, Tumour necrosis factor, Cytotoxicity, Transmission electron microscopy

## Abstract

**Background:**

Acne vulgaris is a chronic skin disorder leading to inflammation as a result of the production of reactive oxygen species due to the active involvement of *Propionibacterium acnes* (*P. acnes*) in the infection site of the skin. The current study was designed to assess the potential of the leaf extract of *Syzygium jambos* L. (Alston) and its compounds for antibacterial and anti-inflammatory activity against the pathogenic *P. acnes*.

**Methods:**

The broth dilution method was used to assess the antibacterial activity. The cytotoxicity investigation on mouse melanocyte (B16-F10) and human leukemic monocyte lymphoma (U937) cells was done using sodium 3’-[1-(phenyl amino-carbonyl)-3,4-tetrazolium]-bis-[4-methoxy-6-nitrobenzene sulfonic acid hydrate (XTT) reagent. The non-toxic concentrations of the samples was investigated for the suppression of cytokines interleukin 8 (IL 8) and tumour necrosis factor (TNF α) by testing the supernatants in the co-culture of the human U937 cells and heat killed *P. acnes* using enzyme immunoassay kits (ELISA). The statistical analysis was done using the Graph Pad Prism 4 program.

**Results:**

Bioassay guided isolation of ethanol extract of the leaves of *S. jambos* led to the isolation of three known compounds namely; squalene, an anacardic acid analogue and ursolic acid which are reported for the first time from this plant. The ethanol extract of *S. jambos* and one of the isolated compound namely, anacardic acid analogue were able to inhibit the growth of *P. acnes* with a noteworthy minimum inhibitory concentration (MIC) value of 31.3 and 7.9 μg/ml, respectively. The ethanol extract and three commercially acquired compounds namely; myricetin, myricitrin, gallic acid exhibited significant antioxidant activity with fifty percent inhibitory concentration (IC_50_) ranging between 0.8-1.9 μg/ml which was comparable to that of vitamin C, the reference antioxidant agent. The plant extract, compounds ursolic acid and myricitrin (commercially acquired) significantly inhibited the release of inflammatory cytokines IL 8 and TNF α by suppressing them by 74 - 99%. TEM micrographs showed the lethal effects of selected samples against *P. acnes*.

**Conclusions:**

The interesting antibacterial, antioxidant and anti-inflammatory effects of *S. jambos* shown in the present study warrant its further investigation in clinical studies for a possible alternative anti-acne agent.

## Background

*Syzygium jambos* L. (Alston) belongs to the family Myrtaceae and is commonly known as rose apple [1] which is widespread in sub-Saharan Africa [2], Central America and Asia [3]. The plant is reported to be used for a variety of ailments and is known for its antipyretic and anti-inflammatory properties. All parts of the plant are reported to have medicinal value. In Indo-China all parts of the plant are used for digestive and tooth ailments. A decoction of the leaves is used as a diuretic, a remedy for sore eyes and for rheumatism. The seeds are used to treat diarrhoea, dysentery, diabetes and catarrh. A decoction of bark is administered to relieve asthma and bronchitis [1].

Previous researchers who have investigated the plant have documented its potential pharmacological value. Acetone and aqueous bark extracts of this plant have been reported to be active against *Staphylococcus aureus*, *Yersinia enterocolitica, Staphylococcus hominis*, *Staphylococcus cohnii* and *Staphylococcus warneri*[4]. In another study, the scientists have investigated the antimicrobial activity of both acetone and aqueous extracts of the leaves, bark and seeds against eight microorganisms namely, *S. aureus*, *Bacillus subtilis*, *Escherichia coli*, *Klebsiella pneumoniae*, *Proteus vulgaris*, *Pseudomonas aeruginosa*, *Salmonella typhi* and *Vibrio cholera.* The acetone bark extract showed growth inhibitory activity against all the microorganisms tested whereas, the leaf extract inhibited only *S. aureus* and the seed extract did not show any inhibitory activity. The aqueous bark extract exhibited growth inhibitory effect against *S. aureus*, *E. coli* and *S. typhi*, whereas, the seed extract inhibited the growth of *P. aeruginosa* and *V. cholerae*, and leaf extract exhibited an inhibitory effect only against *S. typhi*[5]. In a study done by Kuiate et al. [6], it was found that the ethanol bark extract of *S. jambos* and its isolated triterpenoids such as friedelin, β -amyrin acetate, betulinic acid and lupeol exhibited antidermatophytic activity against *Microsporum audouinii*, *Trichophyton mentagrophytes* and *Trichophyton soudanense.*

Acne vulgaris is a chronic inflammatory disorder of the pilosebaceous unit with multifactorial etiology. It affects almost everybody during the course of their life. There are four key processes in the pathogenesis of acne: 1. Increased sebum production, 2. Follicular hyperkeratinization which leads to follicular obstruction, 3. Colonization by the causative agent, *Propionibacterium acnes* and 4. Host inflammatory responses triggered as a results of bacterial infection [7,8].

As a result of the increased sebum production due to high androgen levels, *P. acnes*, a gram positive anaerobic commensal, produces various hydrolytic enzymes that act on the sebum to release free fatty acids. These free fatty acids acts as chemokines and increase the release of pro-inflammatory cytokines like interleukins 8 (IL 8) and tumour necrosis factors (TNF α) which attract macrophages and lead to severe inflammation. Follicular wall ruptures due to the action of hydrolytic enzymes cause oxidative damage with the release of free radicals [7,8]. Therefore, an agent that can inhibit the growth of *P. acnes*, scavenge free radicals and suppress the inflammatory response is promising. The conventional drugs available to treat acne act as antibacterial and anti-inflammatory agents. But these drugs have various side effects such as dryness, itching and hypopigmentation and also have age restrictions. Moreover, bacterial resistance is an ongoing problem. The use of medicinal plants dates back thousands of years. In light of previous reports regarding *S. jambos* as an antimicrobial agent on a variety of micro-organisms, the present study was conducted to explore its efficiency as an anti-acne agent. Thus, the antibacterial activity against *P. acnes*, the antioxidant and anti-inflammatory properties of *S. jambos* and its compounds were investigated.

## Methods

### Chemicals, microbial strain and culture media

Silica gel 60 (70–230 mesh); sephadex LH-20 and all the analytical grade chemicals were purchased from Sigma-Aldrich and Merck SA Pty Ltd. Three chemical compounds namely, myricetin, myricitrin and gallic acid were acquired from Sigma-Aldrich.The ELISA kits were bought from BD Biosciences, Johannesburg, South Africa. Cell proliferation Kit II (XTT) was supplied by Roche diagnostics Pty Ltd., Johannesburg, South Africa. *Propionibacterium acnes* (ATCC 11827) was purchased from Anatech, Johannesburg, South Africa. The cell lines and medium were purchased from Highveld Biological Pty Ltd., Johannesburg, South Africa.

### Plant material

The leaves of *Syzygium jambos* L. (Alston) were collected in August 2010 from the botanical garden of the University of Pretoria. The plant was identified at the H.G.W.J Schweicherdt Herbarium (University of Pretoria, Pretoria) where a voucher, specimen number (PRU 119053), has been deposited for future reference.

### Extraction and purification

**Figure 1:**
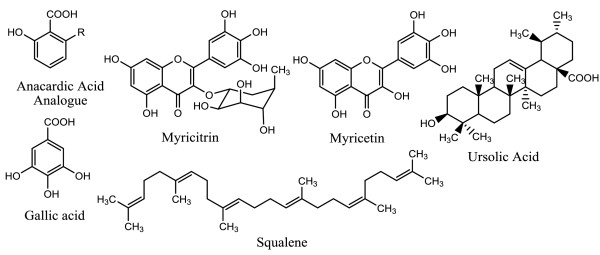
**The chemical structures of the compounds from *Syzygium jambos.***

The air-dried and powdered leaves (1.9 kg) of *S. jambos* were soaked in 7.5 L ethanol for three days at room temperature. The crude ethanol extract (70.5 g) was obtained by concentrating the filtrate under reduced pressure. About 60 g of this ethanol extract was applied to a silica gel column (70 cm × 120 cm) using hexane fractions (Hex): ethyl acetate (EtOAc) of increasing polarity (100:0 to 0:100) followed by 100% methanol (MeOH) as eluents. In total forty three (43) fractions (500 ml) were collected. Similar fractions were combined, according to the thin-layer chromatography (TLC) profiles, which resulted in twelve (12) major fractions (MF). All the twelve (12) major fractions were tested for antibacterial activity against the pathogenic *P. acnes* using the broth dilution method. The results are listed in Table 1. The active MF (2, 4, and 6) were subjected to chromatographic separations to isolate the individual components. MF 2 (200 mg) was applied to a silica gel column using Hex: EtOAc (100:0 to 0:100) gradient as eluents. Eighty five (85) fractions of 50 ml each were collected. The sub-fractions 9–13 were combined on the basis of the TLC analysis which led to the isolation of the compound **1** (10 mg, 0.02%). MF 4 (600 mg) was subjected to a sephadex LH 20 column using 0.5% methanol in dichloromethane (DCM) as the eluent. One hundred and seventeen (117) fractions of 20 ml each were collected. Sub-fractions 80–88 were combined which yielded the compound **2** (8 mg, 0.01%). MF 6 (1.4 g) was subjected to column chromatography similar to MF 4. One hundred (100) fractions of 20 ml each were collected. Sub-fractions 39–56 were combined on the basis of the TLC profiles which yielded the compound **3** (56.5 mg, 0.1%).The chemical structures of all the compounds are illustrated in Figure 1.

**Table 1:** **Antibacterial, antioxidant and cytotoxic effects of ethanol extract of *Syzygium jambos*, fractions and compounds**

**Test samples**	**Antibacterial**	**Antioxidant**		**Cytotoxicity**	
	**MIC μg/ml**	**IC**_**50 **_**μg/ml/(μM)**	**Mg Vit C equivalents/g dry weight**	**B16-F10 mouse melanocytes**	**U937 human macrophage**
				**EC**_**50 **_**μg/ml/(μM)**	
*Syzygium jambos*	31.3	0.9	450.0	60.0	440.0
MF 1,3,5,7-12	^a^Na	^b^-	-	-	-
MF 2	500.0	-	-	-	-
MF 4	62.5	-	-	-	-
MF 6	250.0	-	-	-	-
Squalene	Na	>100	-	>100	>100
Anacardic acid analogue	7.9	>100	-	^c^Nt	57.8
Ursolic acid	Na	>100	-	Nt	38.0/**(83.2)**
Myricetin	Na	0.9/**(3)**	2105.0	11.1/**(35.1)**	19.3/**(60)**
Myricitrin	Na	1.8/**(4)**	1081.0	259.0/**(557.8)**	318.0/**(684.7)**
Gallic acid	Na	0.8/**(4.8)**	2444.9	2.2/(**13.1**)	28.4/**(169)**
^d^PC	3.1/**(7)**	1.9/**(11.3)**	-	4.5 × 10^-3^/**(3.5 × 10**^**6**^**)**	4.5 × 10^-3^/**(3.5 × 10**^**6**^**)**

^a^not active at the highest concentration tested (500 μg/ml).

^b^not applicable: for MF as not tested for antioxidant activity and cytotoxicity, mg equivalent could not be detected for non-antioxidant compounds.

^c^not tested due to low yield of the compound.

^d^positive drug control where tetracycline for antibacterial, vitamin C for antioxidant, actinomycin D for cytotoxicity.

### Antibacterial activity

The ethanol extract and compounds were tested against *P. acnes* by determining the minimum inhibitory concentration (MIC) values obtained by a microdilution method as previously described [9] with few modifications. Briefly, the bacteria was cultured from a Kwik-Stick on nutrient agar and incubated at 37°C for 72 h under anaerobic conditions before the assay. The 72 h culture of the bacteria was dissolved in nutrient broth and the suspension was adjusted to 0.5 McFarland standard turbidity. This resulted in 10^5^-10^6^ colony forming units (CFU)/ml. In a sterile 96-well plate, 100 μl of samples from the stock solution consisting of the plant extract/isolated compounds (2 mg/ml in 10% dimethyl sulphoxide (DMSO)) and the positive control tetracycline (0.2 mg/ml) were diluted with broth. Twofold serial dilutions were made in broth over a range to give concentrations of 500–3.9 μg/ml and 50–0.3 and μg/ml for the plant extract/ isolated compounds and positive control tetracycline, respectively. The bacterial suspension (100 μl) was then added to the wells. The wells with 2.5% DMSO and bacterial suspension without samples served as the solvent and negative controls, respectively. The plates were incubated at 37°C for 72 h in an anaerobic environment. The MIC value was determined by observing colour change in the wells after addition of 2-(4-iodophenyl)-3-(4-nitrophenyl)-5-phenyl (INT) (defined as the lowest concentration that showed no bacterial growth).

### Transmission electron microscopy (TEM)

The TEM procedures followed the protocol of a previous publication [10]. Briefly, bacteria was concentrated by centrifugation at 10 000 rpm for 1 min. The pellet was resuspended in nutrient broth to a final OD550 nm of 1. The concentrations of plant extract were 1.3 and 4 times the MIC; and 5 times the MIC for pure compound in order to visualise the lethal effects of tested samples against bacteria. The bacterial suspension (5 ml) was mixed with plant extract and pure compound to a final concentration of 300 and 100 μg/ml for plant extract and 50 μg/ml for pure compound. Tetracycline (50 μg/ml) and DMSO (2.5%) were used as positive and solvent control, respectively. The pathogen was treated for 72 h; the control group consisted of only bacterial suspension in nutrient broth. Treated and untreated *P. acnes* cultures were centrifuged and fixed in 2.5% glutraldehyde in phosphate buffer at room temperature for 1 h. Samples were washed with phosphate buffer and postfixed in both 1% osmium tetraoxide and uranyl acetate. The cells were dehydrated in ethanol and embedded in quetol resin. Thin sections were prepared with a microtome and micrographs were taken using a JEOL JEM-2100 F field emission electron microscope.

### Antioxidant assay

Antioxidant activity of the ethanol extract of *S. jambos* and compounds was investigated using 1,2-diphenyl-1-picrylhydrazyl (DPPH) antioxidant assay. Following the procedures as described by DuToit et al. [11] for each sample, a dilution series (8 dilutions) was prepared in a 96 well plate by adding distilled water as a dilution medium. Final concentration of the samples ranged from 100 to 0.7 μg/ml. Each concentration was tested in triplicate. Vitamin C was used as a positive control. The radical scavenging capacities of the samples were determined using a BIOTEK Power-wave XS multi well reader (A.D.P., Weltevreden Park, South Africa) to measure the disappearance of DPPH at 550 nm. The radical scavenging activity was measured in terms of the amount of antioxidants necessary to decrease the initial DPPH absorbance by 50% (IC_50_) [11]. The IC_50_ value of each sample was determined graphically by plotting the absorbance of DPPH as a function of the sample concentration in μg/ml. The IC_50_ is the amount of antioxidant necessary to decrease the initial DPPH absorbance by 50%. The results are expressed as the mg vitamin C equivalents/g dry weight and calculated as follows:

$$\begin{array}{ll} \text{VitEAC}\left(\text{mg}\;\text{AA}/100g\right)= & \left({\text{IC}}_{50}\left(\text{vit}\;c\right)/{\text{IC}}_{50}\left(\text{sample}\right)\right)\\ & \times 1000. \end{array}$$

### *In vitro* cytotoxicity assay

The mouse melanocytes (B16-F10) cells were cultured in a complete Minimum Essential Eagle’s Medium (MEM) whereas the human U937 cells were cultured in Roswell Park Memorial Institute (RPMI) containing 10% fetal bovine serum (FBS) and 1% gentamycin. B16-F10 (10^5^ cells per well) and U937 (10^6^ cells per well) were seeded into a 96-well plate. After an overnight incubation at 37°C in 5% CO_2_ and a humidified atmosphere, the extract, compounds and the positive control (actinomycin D) were added to the cells. The final concentrations of plant extract and pure compounds ranged from 400–3.13 μg/ml and 100–1.5 μg/ml, respectively. The highest concentration of positive control (0.05 μg/ml) was serially diluted to eight consecutive wells. The plate was then incubated at 37°C in 5% CO_2_, and a humidified atmosphere after which the toxic effects of the extracts were assayed using the XTT (sodium 3’-[1-(phenyl amino-carbonyl)-3,4-tetrazolium]-bis-[4-methoxy-6-nitrobenzene sulfonic acid hydrate) cytotoxicity assay. Fifty micro litres (50 μl) of XTT reagent (1 mg/ml XTT with 0.383 mg/ml PMS) was added to the wells and incubated for 1 h. The optical densities of the wells were measured at 450 nm (690 nm reference wavelength) using BIOTEK Power-wave XS multi well reader (A.D.P., Weltevreden Park, South Africa). By referring to the control (medium with DMSO), the cell survival rate was assessed. A statistical program (Graph Pad Prism 4) was used to analyze the 50% inhibitory concentration (EC_50_) values.

### Preparation of heat-killed *P. acnes* and the measurement of cytokine production

The effect of selected samples on cytokine production (IL 8 and TNF α) was evaluated using the respective enzyme immunoassay kits (ELISA) by a previously described method [12]. Briefly, the log phase culture of *P. acnes* was harvested, washed three times with phosphate buffer saline (PBS), and incubated at 80°C for 30 min to kill the bacteria. The heat-killed bacteria were stored at 4°C until use. The U937 cells were seeded at 10^6^ cells per well in a 24-well plate and was stimulated with heat killed *P. acnes* (wet weight 100 μg/ml) alone and in combination with the different test samples. Pentoxifylline was used as a control. After 18 h incubation, the cell-free supernatants were collected and the concentrations of IL 8 and TNF α were analysed. Cytokine standards were serially diluted to facilitate the construction of calibration curves necessary for determining protein concentration after treatment with test samples. The ratio (%) of inhibition of the cytokine release was calculated using the following equation:

$$\text{Inhibition}\;\left(\%\right)=100\;\times \;\left(1–T/C\right)$$

Where *T* represents the concentration of the cytokine in the culture supernatant with the test sample, and *C* represents the concentration of cytokine in the culture supernatant with the solvent [13].

### Statistical analysis

All the assays were performed in triplicate. IC_50_ and EC_50_ values for antioxidant and cytotoxicity tests were derived from a nonlinear regression model (curvefit) based on sigmoidal dose response curve (variable) and computed using GraphPad Prism 4 (Graphpad, San Diego, CA, USA).

## Results and discussions

### Identification of isolated compounds

The structure elucidation of the isolated compounds was established on the basis of physical and spectroscopic techniques, especially NMR spectra and direct comparison with spectroscopic measurements to published literature values. The compounds isolated from the ethanol extract of leaves of *S. jambos* were identified as squalene (compound **1**) [14] and ursolic acid (compound **3**; white powder; m.p. 284–286°C) [15]. Compound **2** was obtained as pale yellow liquid. The UV spectrum of compound **2** exhibited maximum absorption (λ_max_) at 243 and 302 nm and in the IR spectrum showed absorption bands at 3448 and 1617 cm^-1^ indicating the characteristics of hydroxybenzoic acid [16]. The ^1^H NMR spectrum of compound **2** showed a triplet at δ 7.40 (J = 8.2), and two doublets at 6.90 (J = 8.2), 6.80 (J = 7.2), and the characteristic signals for a 1, 2, 6-trisubstituted benzene ring. Additionally, the ^13^C NMR spectra showed the signals at δ 176.3 (COOH), 163.5 (C-6), 147.7 (C-2), 135.3 (C-4), 122.7 (C-3) 115.8 (C-1) and 110.3 (C-5), also supported the presence six carbons and a carboxylic group constituting the backbone, although a few signals indicating the presence of long aliphatic chain could not be established. There are many anacardic acids that have been reported by previous researchers which differ in the length of side chain [17,18].Therefore, in the current study compound **2** is being reported as anacardic acid analogue with a side chain R. The ethanol leaves extract of *S. jambos* exhibited significant antibacterial activity against *P. acnes*; therefore, it was decided to acquire three commercially known compounds namely; myricetin (yellow needles; m.p. 356–358°C), myricitrin (white powder; m.p. 206–208°C) and gallic acid (colourless crystals; m.p. 212–214°C) which have been previously isolated from 90% ethanol extract of *S. jambos*[19], to investigate if any of these compounds contribute towards the total extract activity. Also, the presence of these three commercially acquired compounds was confirmed in ethanol extract of *S. jambos* prepared for the current study by TLC.

The present study reports the isolation of squalene, ursolic acid and an analogue of anacardic acid for the first time from *S. jambos*. The presence of the compounds discussed in this study is known since antiquity. Ursolic acid was first time reported in 1920 from epicuticular waxes of apple [20]. Squalene was first reported in 1917 from shark liver oil by Mitsumaru Tsujimoto [21]. Myricetin and myricitrin were first isolated from the bark of *Myrica nagi* and subsequently were reported in the leaves of *Rhus coriaria*, *Myrica gale*, *Pistachia lentiscus* and *Haematoxylon campeachianum*[22,23]. Gallic acid was initially found in the nutgalls of *Rhus toxicodendron* and later in the leaves of the same plant by M. Aschoff and M. Bracconnot in early 19^th^ century [24]. Anacardic acid was first reported from shells of cashew nuts by Stadler in 1847 [25].

### Antibacterial bioassay

The MIC value of the ethanol extract of *S. jambos*, twelve MF and compounds are listed in Table 1. The ethanol extract of *S. jambos* inhibited bacterial growth and exhibited a noteworthy MIC value of 31.3 μg/ml. Anacardic acid analogue was found to be the most active compound against *P. acnes* at MIC value of 7.9 μg/ml as compared to tetracycline (positive control) with MIC value of 3.1 μg/ml. Compounds squalene, ursolic acid, myricetin, myricitrin and gallic acid did not show any inhibitory activity at the highest concentration tested (500 μg/ml).

To the best of our knowledge, the antibacterial activity of *S. jambos* and the compounds (squalene, ursolic acid, myricetin, myricitrin and gallic acid) against *P. acnes* is reported for the first time. In the present study anacardic acid analogue significantly inhibited the growth of *P. acnes*. Our results corroborate well with previous investigations where reports regarding antibacterial activity of a series of synthetic anacardic acids possessing different side-chain lengths against *P. acnes* were found. Four anacardic acids namely; pentadecatrienyl salicylic acid, pentadecadienyl salicylic acid, pentadecenyl salicylic acid and pentadecyl salicylic acid were reported to be active against *P. acnes* with MIC value of 0.78 μg/ml, very similar to our results [18]. In another study, ursolic acid showed MIC value of 4 μg/ml against vancomycin-resistant enterococci [26], myricetin inhibited the growth of methicillin-resistant *S. aureus*, *Burkholderia cepacia* and *K. pneumoniae*[27], different strains of *Staphylococcus epidermidis* exhibited sensitivity towards myricitrin [28] and gallic acid showed growth inhibitory behaviour on *S. aurens*, *Bacillus cereus*, *E. coli* and *Candida albicans*[29]. No scientific report on the antimicrobial activity of squalene was found in the literature.

### Transmission electron microscopy (TEM)

**Figure 2:**
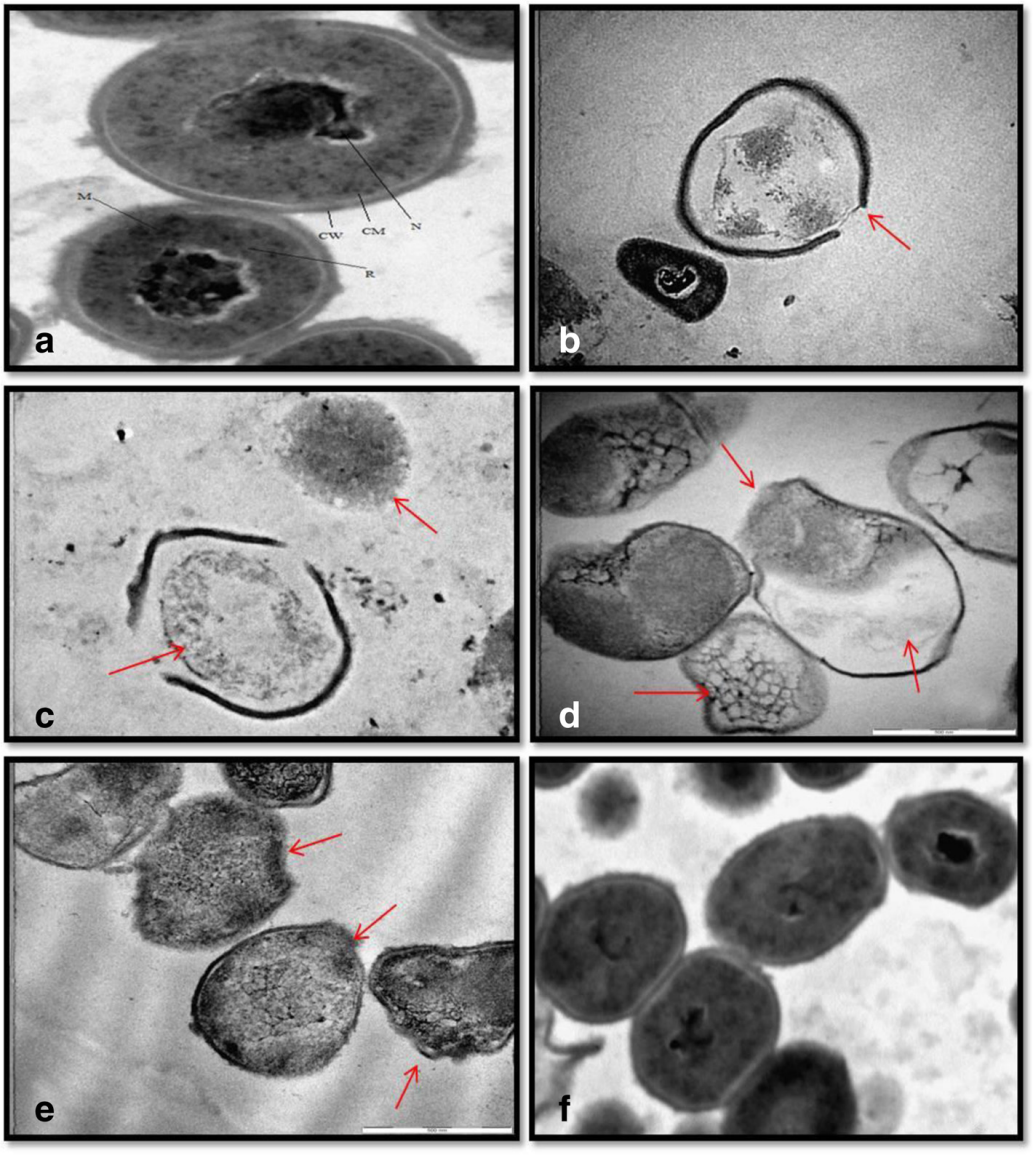
**Transmission electron micrograph of a thin section of *P. acnes *at 60 K magnification. (a)** untreated bacteria, labelled structures: cell wall (CW); cytoplasmic membrane (CM); nucleoid (N); ribosomes (R); mesosomes (M); **(b)***P. acnes* treated with *S. jambos* at 100 μg/ml; **(c)***P. acnes* treated with *S. jambos* at 300 μg/ml; **(d)***P. acnes* treated with anacardic acid analogue at 50 μg/ml; **(e)***P. acnes* treated with positive control (tetracycline) at 50 μg/ml; **(f)***P. acnes* treated with solvent (DMSO at 2.5%). The arrows indicate cell injuries to the *P. acnes*.

For the microscopy studies, the plant extract and isolated compound anacardic acid analogue, which showed inhibitory activity against the bacteria, were selected. The TEM micrograph showed clear differences between untreated and treated *P. acnes*. The untreated *P. acnes* had a distinct cell wall which was long, spindle shaped, smooth and lined with cell membrane. A centrally located nucleoid surrounded by ribosomes was observed (Figure 2a). The TEM micrograph shows cell injury caused to *P. acnes* after treatment with ethanol extract of S*. jambos* for 72 h. *P. acnes* showed breaks in the cell wall when treated with the ethanol extract of *S. jambos* at 100 μg/ml (Figure 2b) while a complete loss of cell wall was also observed at a higher concentration of 300 μg/ml (Figure 2c).The TEM micrographs of *P. acnes* treated with anacardic acid analogue at a concentration of 50 μg/ml showed abnormal changes in cell content material such as shrinkage of intracellular inclusions and the hollow appearance of the bacteria. The outer membrane was found to be irregular and distortions in the cell structure were observed (Figure 2d). Tetracycline at a concentration of 50 μg/ml caused significant damages to the cells of *P. acnes*, leading to damages in the cell membrane, distortion in the cell structure and shrinkage of cell content material (Figure 2e). DMSO at 2.5% exhibited no lethal effects to bacteria (Figure 2f). The TEM micrograph confirms the antibacterial activity of *S. jambos* and bioactive compound (anacardic acid analogue) against *P. acnes.*

### Antioxidant properties of *S. jambos* and its compounds

The DPPH assay indicated the free radical scavenging properties of the samples. Antioxidants are able to stabilize the free DPPH radicals due to their proton donating ability. The scavenging effect of the ethanol extract of *S. jambos* and the compounds (myricetin, myricitrin and gallic acid) on DPPH increased with increasing concentrations. These samples showed significant antioxidant activity with IC_50_ values ranging between 1.9-0.7 μg/ml, very similar to that of Vitamin C, a widely used antioxidant compound exhibiting an IC_50_ value of 1.9 μg/ml. The isolated compounds (squalene, anacardic acid analogue and ursolic acid) did not exhibit any radical scavenging activity at highest concentration tested (100 μg/ml). The results are summarised in Table 1. Our findings were in agreement with previous reports. The DPPH radical scavenging activity of *S. jambos* has been reported previously with IC_50_ value of 14.10 μg/ml [30] and can be explained due to the presence of flavonoids and polyphenol compounds namely; myricetin, myricitrin and gallic acid. The antioxidant activity of flavonoids and polyphenols is ascribed to the presence of free hydroxyl (-OH) substitutes. Myricetin possess six free hydroxyl radicals at 3, 5, 7, 3΄, 4΄, 5΄ carbon positions and gallic acid possess three free hydroxyl radicals at 3, 4, 5 carbon positions. In the current study, ursolic acid did not demonstrate any antioxidant activity even at its highest concentration of 100 μg/ml. Similar to our findings, another researcher found ursolic acid to be inactive in inhibiting the generation of free radicals at concentrations of 0.25 and 0.5 mg/ml [31]. In our study squalene did not show any DPPH free radical scavenging activity and this can be explained as it is a single oxygen scavenger and lacks free hydroxyl groups, therefore cannot scavenge a DPPH radical [32]. In the current study, no antioxidant activity for anacardic acid analogue was found. Similar to our findings, 6-pentadecenylsalicylic acid isolated from *Anacardium occidentale* did not exhibit notable DPPH radical scavenging activity [33].

### Effect of ethanol extract of *S. jambos* and its compounds on the cell viability

The cytotoxicity assay of the extracts and the compounds was done on B16-F10 mouse melanocyte and U937 human macrophage cells. Due to the low yield, no further test could be done on the isolated compound anacardic acid analogue. All the results are listed in Table 1. To the best of our knowledge, the cytotoxicity results of ethanol extract of *S. jambos* and the compounds (squalene, ursolic acid, myricetin, myricitrin and gallic acid) on the cell viability of B16-F10 and U937 cells obtained in the present study are reported for the first time. *S. jambos* exhibited moderate toxicity to B16-F10 cells and no toxicity to U937 cells. In contrary, previous studies have reported strong cytotoxic effects of 70% acetone extract of *S. jambos* on human leukemia cells (HL-60) with an IC_50_ value of 10.2 μg/ml [34]. In the current study, squalene was not found to be toxic on both the cell lines with 100% viability of cells even at highest concentration of 100 μg/ml tested. Similar to our findings, squalene was reported to be non-toxic to human mammary epithelial cells (MCF10A) [35]. In the present study, ursolic acid was found to be moderately toxic to U937 cells. Contrary to our findings, in a study, significant cytotoxic effects of ursolic acid against lymphocytic leukemia cells (P 388 and L 1210), human colon cells (HCT 8) and mammary (MCF 7) tumour cells were reported [36]. Myricetin and gallic acid showed significant toxicity to B16-F10 cells and moderate toxicity on U937 cells. Similar to our results, myricetin showed strong toxicity to human A549 lung cells [37] and gallic acid was not reported to be toxic on human lymphocytes derived from fresh blood [38]. In our study myricitrin did not show any toxicity to either of the cell lines and similar results are reported in a study where myricitrin was not found to be toxic on murine fibro sarcoma (L929) cells [39].

### Effect of ethanol extract of *S. jambos* and its compounds on the pro-inflammatory cytokines

*P. acnes* contribute to the inflammatory nature of acne by inducing macrophages to secrete pro-inflammatory cytokines like IL 8 and TNF α. In the current study, U937 cells treated with heat-killed *P. acnes* resulted in an increase in secretion of IL 8 and TNF α (data not shown). These results confirmed that *P. acnes* are capable in eliciting the inflammatory response which plays an important role in acne pathogenesis.

For the interpretation of the results, the percentage values are classified under four groups; an inhibition between 100 and 70% was accepted as high, between 69 and 40% as moderate, between 39 and 20% as low. An inhibition of less than 20% was considered to be insignificant.

To test the anti-inflammatory effects of *S. jambos* and its compounds, *in vitro* screening at three non-cytotoxic concentrations of the samples to the cells were applied. As shown in Table 2, the ethanol extract of *S. jambos* and the compounds (ursolic acid, myricetin, myricitrin and gallic acid) decreased the production of IL 8 and TNF α in a dose-dependent manner. A significant inhibition of IL 8 and TNF α was observed for the *S. jambos* extract*,* ursolic acid and myricitrin at their highest concentrations. Gallic acid, although suppressed TNF α drastically, had no significant suppression of IL 8 at its highest concentration. Myricetin moderately decreased the release of TNF α and showed low inhibition of IL 8 at the highest concentration tested. Pentoxifylline, which was used as a control, behaved differently on the cytokines. Based on previous reports, it down regulated the secretion of TNF α and caused no change in the IL 8 released [40]. As shown in Table 2, our results corroborate well with previous investigations. Very high inhibition of TNF α was observed at 100 and 50 μg/ml of pentoxifylline whereas no significant change in IL 8 concentration was observed. Furthermore, the test samples did not increase the secretion of the cytokines in the culture of U937 cells in the absence of the heat killed *P. acnes* (data not shown). Other researchers have previously reported the anti-inflammatory potential of the samples isolated in this study. Myricetin was reported to inhibit the release of IL 8 and TNF α from human umbilical cord blood-derived cultured mast cells [41], myricitrin and myricetin suppressed TNF α production in LPS/IFN-γ stimulated J774.A1 cell line [42]. Gallic acid inhibited the production of IL 8 and TNF α from *Fusobacterium nucleatum* activated human mouth epithelial cell line and human mast cells, respectively [43,44]. Ursolic acid inhibited IL 8 secretion from HT29 cells [45]. To the best of our knowledge, no reports about *S. jambos* in context with suppression of cytokines were found. Although, similar to our results, other plants such as *Eucommia ulmoides* and *Ilex paraguariensis* extracts were reported to reduce the secretion of IL 8 and TNF α in human monocytic THP-1 cells pre-treated with *P. acnes* at concentration of 0.1 mg/ml [12].

**Table 2:** **Anti-inflammatory effects of ethanol extract of *Syzygium jambos *** **and the compounds**

**Test samples**	**Final concentration μg/ml/(μM)**	**Inhibitory ratio (%)**	
		**IL 8**	**TNF α**
*Syzygium jambos*	100	85.0	99.7
	50	69.8	79.9
	10	23.9	28.6
Ursolic acid	25/**(54)**	88.3	74.0
	12.5/**(27)**	78.9	59.8
	6.2/**(13)**	53.2	14.0
Myricetin	12.5/**(39)**	34	44.1
	6.2/**(19)**	10	27.1
	3.1/**(9)**	-0.5	-4.8
Myricitrin	100/**(215)**	99.6	99.7
	50/**(107)**	94.3	99.7
	10/**(21)**	40.6	32.8
Gallic acid	25/**(146)**	29.9	84.9
	12.5/**(73)**	19.5	68.0
	6.2/**(36)**	7.6	20.1
Pentoxifylline	100/**(359)**	-1.3	99.7
	50/**(179)**	-5.0	99.7
	10/**(35)**	-0.16	66.8

The anti-inflammatory activity and release of cytokines like IL 8 and TNF α is linked with an inflammatory mediator nuclear factor-kappa B (NF-κB). NF-κB is a transcription factor that resides in the cytoplasm of every cell and its constitutive activation is linked with *P. acnes* infection. The suppression of the cytokines discussed in this study can possibly be due to blocking activation of common transcription factor such as NF-κB involved in their induction.

## Conclusion

The present study provided an important insight on the plants and compounds to be promising source of alternative medicine. Effective anti-acne agents possess three essential capabilities of antibacterial, antioxidant and anti-inflammatory activities. The experimental data gathered herein shows significant antibacterial, antioxidant and anti-inflammatory activities of the ethanol extract of *S. jambos* which might be due to the synergistic action of compounds present in it. Additionally, the plant extract was not found to be toxic to human cells. Therefore, *S. jambos* could be an ideal concomitant for an alternative anti-acne agent. This study will be helpful to understand this important herbal medicine and further clinical trials are under way.

## Abbreviations

AA: Antioxidant activity; B16-F10: Mouse melanocytes; DPPH: 2, 2-diphenyl-1-picrylhydrazyl; DMSO: Dimethyl sulphoxide; ELIZA: Enzyme linked immunosorbent assay; FBS: Fetal bovine serum; IL 8: Interleukin 8; INT: 2-(4-iodophenyl)-3-(4-nitrophenyl)-5-phenyl; MF: Major fraction; MEM: Minimal essential eagle’s medium; MIC: Minimum inhibitory concentration; NMR: Nuclear magnetic resonance; OD: Optical density; RPMI: Roswell Park Memorial Institute; TEM: Transmission electron microscopy; TLC: Thin layer chromatography; TNF α: Tumour necrosis alpha; UV: Ultra violet light; U937: Human leukemic monocyte lymphoma; VitEAC: Vitamin C equivalents/g dry weight; XTT: Sodium 3’-[1-(phenyl amino-carbonyl)-3,4-tetrazolium]-bis-[4-methoxy-6-nitrobenzene sulfonic acid hydrate.

## Competing interests

The authors declare that they have no competing interests.

## Authors’ contributions

RS conceived the study, carried out the experimentation, drafted the manuscript. NK and AH supervised the isolation, did all the characterisation of the compounds, edited the manuscript, NL supervised the project and edited the manuscript. All the authors read and approved the final manuscript.

## Pre-publication history

The pre-publication history for this paper can be accessed here:

http://www.biomedcentral.com/1472-6882/13/292/prepub

## References

[B1] Lim TK Myrtaceae, *Syzygium jambos* Edible Medicinal and Non-medicinal Plants: Fruits. Volume 3 2012 London, New York: Dordrecht Heidelberg 765

[B2] Adjanohoun EJ Contribution auxetudes ethnobotaniques et floristiques en Republique Populaire du Benin 1989 France: Medecine traditionnelle et pharmacopee. Agence de cooperation culturelle et technique

[B3] Maskey K Shah BB Sugars in some Nepalese edible wild fruits J Nepal Chemical Soc 1982 2 23 30

[B4] Djipa CD Delmee M Quetin-Leclercq J Antimicrobial activity of bark extracts of *Syzygium jambos* (L.) Alston (Myrtaceae) J Ethnopharmacol 2000 17 307 313 10904178 10.1016/s0378-8741(99)00186-5

[B5] Murugan S Uma Devi P Parameshwari KN Mani KR Antimicrobial activity of *Syzygium jambos* against selected human pathogens Int J Pharm Pharmaceut Sci 2011 3 44 47

[B6] Kuiate JR Mouokeu S Wabo HK Tane P Antidermatophytic triterpenoids from *Syzygium jambos* (L.) Alston (Myrtaceae) Phytother Res 2007 21 149 152 10.1002/ptr.2039 17128435

[B7] Shaw L Kennedy C The treatment of acne J Paediatr Child Health 2007 17 385 389 10.1016/j.paed.2007.07.005

[B8] Arican O Kurutas EB Sasmaz S Oxidative stress in patients with acne vulgaris Mediators Inflamm 2005 6 380 384 16489259 10.1155/MI.2005.380 PMC1533901

[B9] Mapunya MB Hussein AA Rodriguez B Lall N Tyrosinase activity of *Greyia flanaganii* Bolus constituents Phytomedicine 2011 18 1006 1012 10.1016/j.phymed.2011.03.013 21680165

[B10] Pan CY Chen JY Lin TL Lin CH *In vitro* activities of three synthetic peptides derived from epinecidin-1 and an anti-lipopolysaccharide factor against *Propionibacterium acnes*, *Candida albicans*, and *Trichomonas vaginalis* Peptides 2009 30 1058 1068 10.1016/j.peptides.2009.02.006 19463737

[B11] Du Toit R Volsteedt Y Apostolides Z Comparison of the antioxidant content of the fruits, vegetables and teas measured as Vitamin C equivalents Toxicology 2001 166 63 69 10.1016/S0300-483X(01)00446-2 11518612

[B12] Tsai TH Tsai TH Wu WH Tseng JP Tsai PJ *In vitro* antimicrobial and anti-inflammatory effects of herbs against *Propionibacterium acnes* Food Chem 2010 119 964 968 10.1016/j.foodchem.2009.07.062

[B13] Yesilada E Ustun O Sezik E Takaishi Y Ono Y Honda G Inhibitory effects of Turkish folk remedies on inflammatory cytokines: interleukin-1α, interleukin-1β and tumour necrosis factor α J Ethnopharmacol 1997 58 59 73 10.1016/S0378-8741(97)00076-7 9324006

[B14] Tchinda AT Teshome A Dagne E Arnold N Wessjohann LA Squalene and amentoflavone from *Antidesma laciniatum* Bull Chem Soc Ethiop 2006 20 325 328

[B15] Guvenalp Z Kilic N Kazaz C Kaya Y Demirezer LO Chemical Constituents of *Galium tortumense* Turk J Chem 2006 30 515 523

[B16] Yalpani M Tyman JP The phenolic acids of *Pistachia vera* Phytochemistry 1983 22 2263 2266 10.1016/S0031-9422(00)80158-2

[B17] Liua Y Abreu PJM Long Chain Alkyl and Alkenyl Phenols from the Roots of *Ozoroa insignis* J Braz Chem Soc 2006 17 527 532 10.1590/S0103-50532006000300015

[B18] Kubo I Muroi H Himejima M Structure-Antibacterial Activity Relationships of Anacardic Acids J Agric Food Chem 1993 41 1016 1019 10.1021/jf00030a036

[B19] Jayprakasham R Chapter 5- Phytochemical examination of Syzygium jambos 2010 192 213 http://shodhganga.inflibnet.ac.in/bitstream/10603/961/10/10_chapter%205.pdf

[B20] Belding RD Blankenship SM Young E Composition and variability of epicuticular waxes in apple cultivars J Am Soc Hortic Sci 1998 123 348 356

[B21] Tsujimoto M Saturated hydrocarbons in basing-shark liver oil J Ind Eng Chem 1917 9 1098 10.1021/ie50096a013

[B22] Perkin AG Myricetin Part II J Chem Soc 1902 81 203 Transactions

[B23] Wurdack JH The natural plant coloring matters J Am Pharm Assoc 1924 13 307 315

[B24] Brewster D The London and Edinburgh Philosophical Magazine and Journal of Science 1837 London: R. And J.E. Taylor Press 163

[B25] Harvey MT Caplan S Cashew Nutshell liquid J Ind Eng Chem 1940 32 1309

[B26] Horiuchi K Shiota S Hatano T Yoshida T Kuroda T Tsuchiya T Antimicrobial activity of oleanolic acid from *Salvia officinalis* and related compounds on vancomycin-resistant enterococci (VRE) Biol Pharm Bull 2007 30 1147 1149 10.1248/bpb.30.1147 17541170

[B27] Xu HX Lee SF Activity of plant flavonoids against antibiotic resistant bacteria Phytother Res 2001 15 39 43 10.1002/1099-1573(200102)15:1<39::AID-PTR684>3.0.CO;2-R 11180521

[B28] Pistelli L Bertoli A Noccioli C Mendez J Musmanno RA Maggio TD Coratza G Antimicrobial activity of *Inga fendleriana* extracts and isolated flavonoids Nat Prod Commun 2009 4 1679 1683 20120106

[B29] Panizzi L Caponi C Catalano S Cioni PL Morelli I In vitro antimicrobial activity of extracts and isolated constituents of *Rubus ulmifolius* J Ethnopharmacol 2002 79 165 168 10.1016/S0378-8741(01)00363-4 11801377

[B30] Islam MR Parvin MS Islam ME Antioxidant and hepatoprotective activity of an ethanol extract of *Syzygium jambos* (L.) leaves Drug Discov Ther 2012 6 205 211 23006991

[B31] Jung HA Park JC Chung HY Kim J Choi JS Antioxidant flavonoids and chlorogenic acid from the leaves of *Eriobotrya japonica* Arch Pharm Res 1999 22 213 218 10.1007/BF02976549 10230515

[B32] Ko TF Weng YM Chiou RY Squalene content and antioxidant activity of *Terminalia catappa* leaves and seeds J Agric Food Chem 2002 50 5343 5348 10.1021/jf0203500 12207472

[B33] Kubo I Masuoka N Ha TJ Tsujimoto K Antioxidant activity of anacardic acids Food Chem 2006 99 555 562 10.1016/j.foodchem.2005.08.023

[B34] Yang LL Lee CY Yen KY Induction of apoptosis by hydrolyzable tannins from *Eugenia jambos* L. on human leukemia cells Cancer Lett 2000 157 65 75 10.1016/S0304-3835(00)00477-8 10893444

[B35] Warleta F Campos M Allouche Y Sanchez-Quesada C Ruiz-Mora J Baltran G Gaforio JJ Squalene protects against oxidative DNA damage in MCF10A human mammary epithelial cells but not in MCF7 and MDA-MB-231 human breast cells Food Chem Toxicol 2010 48 1092 1100 10.1016/j.fct.2010.01.031 20138105

[B36] Lee KH Lin YM Wu TS Zhang DC Yamagishi T Hayashi T Hall IH Chang JJ Wu RY Yang TH The cytotoxic principles of *Prunella vulgaris*, *Psychotria serpens* and *Hyptis capitata*: ursolic acid and relative derivatives Planta Med 1988 54 308 311 10.1055/s-2006-962441 3222376

[B37] Lu J Papp LV Fang J Nieto SR Zhivotovsky B Holmgren A Inhibition of mammalian thioredoxin reductase by some flavonoids: implications for myricetin and quercetin anticancer activity Cancer Res 2006 66 4410 4418 10.1158/0008-5472.CAN-05-3310 16618767

[B38] Yen GC Duh PD Tsai HL Antioxidant and pro-oxidant properties of ascorbic acid and gallic acid Food Chem 2002 79 307 313 10.1016/S0308-8146(02)00145-0

[B39] Hsu LW Chang SC Shen CH Liao YX Chuang KS Flavone derivatives as TNF alpha inhibitors or antagonists 2004 US Patent Application number 10/992,178

[B40] D’Hellencourt CL Diaw L Cornillet P Guenounou M Differential regulation of TNFα, IL-1β, IL-6, IL-8, TNFβ and IL-10 by pentoxifylline Int J Immunopharmac 1996 18 739 748 10.1016/S0192-0561(97)85556-7 9172017

[B41] Kempuraj D Madhappan B Christodoulou S Boucher W Cao J Papadopoulou N Cetrulo CL Theoharides TC Flavonols inhibit proinflammatory mediator release, intracellular calcium ion levels and protein kinase C theta phosphorylation in human mast cells Br J Pharmacol 2005 145 934 944 10.1038/sj.bjp.0706246 15912140 PMC1576204

[B42] Ferreria LC Guimaraes AG Paula CA Michel MCP Guimaraes RG Rezende SA Filho JDS Guimaraes DAS Anti-inflammatory and antinociceptive activities of *Compomanesia adamantium* J Ethnopharmacol 2013 145 100 108 10.1016/j.jep.2012.10.037 23123269

[B43] Kang MS Jang HS Oh JS Yang KH Choi NK Lim HS Kim SM Effects of methyl gallate and gallic acid on the production of inflammatory mediators interleulin-6 and interleulin-8 by oral epithelial cells stimulated with *Fusobacterium nucleatum* J Microbiol 2009 47 760 767 10.1007/s12275-009-0097-7 20127471

[B44] Kim SH Jun CD Suk K Choi BJ Lim H Park S Lee SH Shin HY Kim DK Shin TY Gallic acid inhibits histamine release and pro-inflammatory cytokine production in mast cells Toxicol Sci 2006 91 123 131 10.1093/toxsci/kfj063 16322071

[B45] Thuong PT Jin WY Lee JP Seong RS Lee YM Seong YH Song KS Bae KH Inhibitory effect on TNF- α induced IL-8 production in the HT29 cell of constituents from the leaf and stem of *Weigela subsessilis* Arch Pharm Res 2005 28 1135 1141 10.1007/BF02972975 16276968

